# Metformin use and mortality in Asian, diabetic patients with prostate cancer on androgen deprivation therapy: A population‐based study

**DOI:** 10.1002/pros.24443

**Published:** 2022-09-30

**Authors:** Yan Hiu Athena Lee, Jeremy Man Ho Hui, Jeffrey Shi Kai Chan, Kang Liu, Edward C. Dee, Kenrick Ng, Pias Tang, Gary Tse, Chi Fai Ng

**Affiliations:** ^1^ Diabetes Research Unit, Cardiovascular Analytics Group, Hong Kong, China‐UK Collaboration Hong Kong China; ^2^ Department of Surgery, Division of Urology, Faculty of Medicine The Chinese University of Hong Kong Hong Kong China; ^3^ Department of Radiation Oncology Memorial Sloan Kettering Cancer Center New York New York USA; ^4^ Department of Medical Oncology University College London Hospitals NHS Foundation Trust London UK; ^5^ Tianjin Key Laboratory of Ionic‐Molecular Function of Cardiovascular Disease, Department of Cardiology, Tianjin Institute of Cardiology Second Hospital of Tianjin Medical University Tianjin China; ^6^ Kent and Medway Medical School, Canterbury Kent UK; ^7^ SH Ho Urology Centre The Chinese University of Hong Kong Hong Kong China

**Keywords:** androgens, cohort studies, diabetes mellitus, metformin, prostatic neoplasms

## Abstract

**Background:**

This study aims to examine the associations between metformin use concurrent with androgen deprivation therapy (ADT) and mortality risks in Asian, diabetic patients with prostate cancer (PCa).

**Methods:**

This study identified diabetic adults with PCa receiving any ADT attending public hospitals in Hong Kong between December 1999 and March 2021 retrospectively, with follow‐up until September 2021. Patients with <6 months of medical castration without subsequent bilateral orchidectomy, <6 months of concurrent metformin use and ADT, or missing baseline HbA1c were excluded. Metformin users had ≥180 days of concurrent metformin use and ADT, while non‐users had no concurrent metformin use and ADT or never used metformin. The primary outcome was PCa‐related mortality. The secondary outcome was all‐cause mortality. The study used inverse probability treatment weighting to balance covariates.

**Results:**

The analyzed cohort consisted of 1971 patients (1284 metformin users and 687 non‐users; mean age 76.2 ± 7.8 years). Over a mean follow‐up of 4.1 ± 3.2 years, metformin users had significantly lower risks of PCa‐related mortality (weighted hazard ratio [wHR]: 0.49 [95% confidence interval, CI:  0.39–0.61], *p* < 0.001) and all‐cause mortality (wHR 0.53 [0.46–0.61], *p* < 0.001), independent of diabetic control or status of chronic kidney disease. Such effects appeared stronger in patients with less advanced PCa, which is reflected by the absence of androgen receptor antagonist or chemotherapy use (*p* value for interaction: 0.017 for PCa‐related mortality; 0.048 for all‐cause mortality).

**Conclusions:**

Metformin use concurrent with ADT was associated with lower risks of mortality in Asian, diabetic patients with PCa.

## 1 INTRODUCTION

Globally, prostate cancer (PCa) was the second most common cancer and fifth major cause of cancer mortality among males in 2012.^1^ Patients with PCa have a high rate of mortality, which could be partially attributed to the treatment they receive. Androgen deprivation therapy (ADT) has been the mainstay treatment of locally advanced and metastatic PCa. Despite the benefits associated with ADT, it can cause a range of side effects such as metabolic changes and greater risk of diabetes mellitus (DM) and cardiovascular diseases.^2^, ^3^ Metformin is used as first‐line pharmacotherapy to treat people with DM. In addition, it could decrease some of the unfavorable metabolic consequences of ADT. Moreover, metformin could enhance the tumor‐suppressive effect of ADT,^4^ possibly because of its anticancer activity and interplay with the androgen receptor (AR) signaling axis.^5^ It has been shown that metformin may decrease risk of biochemical recurrence^6^ and improve survival in patients with PCa.^7^, ^8^ However, it is unclear whether the survival benefits associated with metformin use in PCa is applicable to ADT. Research in this area is important, especially in Asian populations where the incidence of PCa is increasing.^9^ Therefore, this study examined the associations between metformin use concurrent with ADT and mortality risks among Asian, diabetic patients with PCa.

## 2 MATERIALS AND METHODS

### 2.1 Source of data

This retrospective cohort study was performed in accordance with the Declaration of Helsinki and the STROBE guideline^10^ and has been approved by the Joint Chinese University of Hong Kong–New Territories East Cluster Clinical Research Ethics Committee. The requirement for individual patients' consent has been waived due to the use of retrospective data. All data underlying this study are available upon reasonable request to the corresponding author.

All data used in this study were retrieved from the Clinical Data Analysis and Reporting System (CDARS), a population‐based electronic health records database documenting key demographics, diagnoses, procedures, and medication records of all patients who attend public healthcare institutions in Hong Kong. All diagnoses are coded by the *International Classification of Diseases, Ninth Revision* (ICD‐9) codes. CDARS is linked to the Hong Kong Death Registry, a population‐wide governmental registry of all Hong Kong citizens' death records, from which mortality data may be obtained. Causes of mortality were encoded using either ICD‐9 or ICD‐10 codes, depending on the year of death. This system has been used extensively for research.^11^, ^12^, ^13^, ^14^

### 2.2 Study design and population

Adult patients (18 years old or above) diagnosed with PCa and DM, who were receiving ADT in Hong Kong between December 1, 1999 and March 31, 2021 were included. Diagnosis of PCa was determined by ICD‐9 codes (Supporting Information: Table 1), while that of DM was determined by the corresponding ICD‐9 codes (Supporting Information: Table 1), any baseline use of antidiabetic medication, or hemoglobin A1c (HbA1c) level higher than 6.5% before the initiation of ADT. ADT included bilateral orchidectomy, gonadotrophin‐releasing hormone agonists, and gonadotrophin‐releasing hormone antagonists.

The following patients were excluded: (a) with less than 6 months of medical castration without subsequent bilateral orchidectomy, (b) with less than 6 months of concurrent metformin use and ADT, and (c) with missing baseline HbA1c value.

Metformin users were defined as patients who had at least 6 months of concurrent metformin use and ADT. Metformin non‐users were defined as patients without concurrent metformin use and ADT or without any metformin use.

### 2.3 Follow‐up and outcomes

All patients were followed‐up from the day of ADT initiation (baseline date) until September 30, 2021. The primary outcome was PCa‐related mortality. The secondary outcome was all‐cause mortality. The duration between ADT initiation and mortality was recorded. All causes of death were determined by ICD codes (Supporting Information: Table 1).

### 2.4 Statistical analyses

All patients' age and other comorbidities at baseline, as determined by ICD‐9 codes (Supporting Information: Table 2), type of ADT received, use of other medications, use of other treatments of PCa (radiotherapy, radical prostatectomy, prior chemotherapy and chemotherapy concurrent with ADT), and HbA1c level at baseline were recorded. The list of medications used were summarized in Supporting Information: Table 3.

Continuous variables were expressed as mean ± SD. Inverse probability treatment weighting (IPTW) using the aforementioned covariates was used to balance the treatment groups. Standardized mean differences (SMDs) were calculated for each covariate to examine the balance of covariates between treatment groups, with values ≤0.1 being considered to represent good balance.

IPTW univariable Cox regression was used to assess the associations of metformin treatment with the risks of outcomes. Weighted hazard ratios (wHR) with 95% confidence intervals (CI) were used as the summary statistics. Kaplan–Meier curves were used to visualize the cumulative freedom from the outcomes.

All *p* values were two‐sided, with values less than 0.05 considered statistically significant. All statistical analyses were performed on SPSS (version 25.0, IBM Corp.) or Stata (Version 13.0, StataCorp LLC).

### 2.5 Subgroup analyses

An a priori subgroup analysis was performed for the use of AR antagonists or chemotherapy, typical treatments of metastatic PCa, as surrogate markers of metastatic PCa. A second a priori subgroup analysis was performed for each type of ADT given to investigate whether the associations between metformin use and mortality risks remained significant for different types of ADT. Furthermore, to examine the interactions between metformin's associations with mortality risks and diabetic control, for which baseline insulin use and baseline HbA1c level were used as surrogate markers, two a priori subgroup analyses were performed for these two covariates, with correspondin*g p* values for interaction generated.

### 2.6 Sensitivity analyses

As chronic kidney disease (CKD) is a contraindication for metformin prescription, a sensitivity analysis that only included patients without CKD at baseline was performed. To investigate whether the observed results were affected by metformin use that was not concurrent with ADT, a second sensitivity analysis was performed where patients who had any metformin use at any timepoint were excluded from the metformin non‐user group, such that metformin users were compared only against patients who never used metformin. To further investigate whether metformin use at the time of ADT initiation had any effect on the observed results, a third sensitivity analysis was performed where patients who were not using metformin at the time of ADT initiation were excluded from the metformin user group, and patients who had any metformin use at any timepoint were excluded from the metformin non‐user group, such that only metformin users who had metformin use at the time of ADT initiation were compared against patients who never used metformin.

To investigate the association between the duration of concurrent metformin use and ADT (metformin use duration) and the risk of the outcomes, unweighted multivariable Cox regression was performed on patients in the metformin group. Backward selection Cox regression was performed, initially entering all recorded covariates as aforementioned, and with *p* ≥ 0.05 as the threshold for variable removal. A fractional polynomial curve was constructed for each outcome with adjustment for the covariates in the final model as obtained by the backward selection Cox regression above, visualizing the association between metformin use duration and the risk of outcomes across the observed range of the former.

To account for erroneous estimation of hazards by conventional survival analyses brought by high mortality rate, competing risk analysis was performed with non‐PCa‐related mortality as the competing event using Fine‐Gray subdistribution model. Univariable competing risk regression with IPTW was used to assess the association between metformin use and risk of PCa‐related mortality. Subhazard ratios (SHRs) with 95% CI were used as summary statistics.

## 3 RESULTS

### 3.1 Study cohort

**Figure 1:**
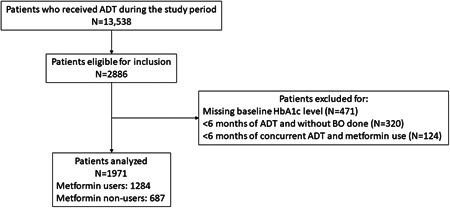
Study flow chart. ADT, androgen deprivation therapy. BO, bilateral orchidectomy. HbA1c, hemoglobin A1c.

In total, 2886 patients were eligible for inclusion. After applying the exclusion criteria, 1971 patients were included in the analysis (Figure 1), of whom 1284 were metformin users and 687 were non‐users. The mean age was 76.2 ± 7.8 years; the mean metformin duration was 10.0 ± 5.1 years; 652 patients (33.1%) received bilateral orchidectomy only, 1090 (55.3%) received medical castration only, and 229 (11.6%) received both. Among those who received medical castration only, the mean duration of treatment was 2.9 ± 2.3 years. Baseline characteristics of included patients were summarized in Table 1, which also demonstrates good balance of all covariates by IPTW (SMD ≤ 0.1 for all). The comparison of baseline characteristics between patients included and excluded in the analysis is shown in Supporting Information: Table 4, with higher percentage for the use of baseline chemotherapy, chemotherapy concurrent with ADT, and AR antagonists among patients to be analyzed.

**Table 1:** Baseline characteristics with standardized mean differences (SMD) before and after inverse probability treatment weighting (IPTW)

	Metformin non‐users (*N* = 687)	Metformin users (*N* = 1284)	Unweighted SMD	SMD with IPTW
Age, years	78.3 ± 7.8	75.1 ± 7.7	0.42	0.02
Use of GnRH agonist or antagonist, *n* (%)	452 (65.8)	867 (67.5)	0.04	0.06
Bilateral orchidectomy, *n* (%)	321 (46.7)	561 (43.7)	0.06	0.05
Hypertension, *n* (%)	415 (60.4)	580 (45.1)	0.31	0.01
Ischemic heart disease, *n* (%)	194 (28.2)	222 (17.3)	0.26	0.01
Myocardial infarction, *n* (%)	75 (10.9)	47 (3.7)	0.28	0.01
Heart failure, *n* (%)	108 (15.7)	84 (6.5)	0.29	<0.01
Stroke or transient ischemic attack, *n* (%)	144 (21.0)	152 (11.8)	0.25	0.02
Chronic kidney disease, *n* (%)	109 (15.9)	35 (2.7)	0.46	<0.01
Anemia, *n* (%)	108 (15.7)	111 (8.6)	0.22	<0.01
Atrial fibrillation, *n* (%)	61 (8.9)	72 (5.6)	0.13	<0.01
Chronic liver disease, *n* (%)	15 (2.2)	29 (2.3)	<0.01	<0.01
Chronic obstructive pulmonary disease, *n* (%)	39 (5.7)	58 (4.5)	0.05	0.05
Hyperlipidemia, *n* (%)	170 (24.7)	267 (20.8)	0.09	<0.01
Ever underwent radiotherapy, *n* (%)	116 (16.9)	238 (18.5)	0.04	0.03
Ever underwent radical prostatectomy, *n* (%)	233 (33.9)	405 (31.5)	0.05	<0.01
Any malignancy, n (%)	104 (15.1)	151 (11.8)	0.10	0.01
ACEI/ARB use, *n* (%)	402 (58.5)	821 (63.9)	0.11	0.04
Beta‐blocker use, *n* (%)	403 (58.7)	571 (44.4)	0.29	0.02
Dihydropyridine calcium channel blocker use, *n* (%)	492 (71.6)	796 (61.9)	0.21	<0.01
Insulin use, *n* (%)	222 (32.3)	279 (21.7)	0.24	0.03
Statin use, *n* (%)	419 (61.0)	763 (59.4)	0.03	0.02
Corticosteroid use, *n* (%)	153 (22.3)	203 (15.8)	0.17	0.08
Antiplatelet use, *n* (%)	335 (48.8)	442 (34.4)	0.30	0.01
Anticoagulant use, *n* (%)	50 (7.3)	65 (5.1)	0.09	<0.01
Androgen receptor antagonist use, *n* (%)	257 (37.4)	579 (45.1)	0.15	0.02
Prior chemotherapy, *n* (%)	3 (0.4)	8 (0.6)	0.03	0.02
Chemotherapy concurrent with ADT, *n* (%)	36 (5.2)	129 (10.0)	0.18	0.03
HbA1c, %	6.7 ± 1.2	7.2 ± 1.3	0.34	0.10

Abbreviations: ACEI, angiotensin‐converting enzyme inhibitor; ADT, androgen deprivation therapy; ARB, angiotensin receptor blocker; GnRH, gonadotropin hormone‐releasing hormone; HbA1c, hemoglobin A1c.

### 3.2 Outcomes

Over a mean follow‐up duration of 4.1 ± 3.2 years, 479 patients (24.3%) had PCa‐related mortality and 1226 (62.2%) had all‐cause mortality.

**Figure 2:**
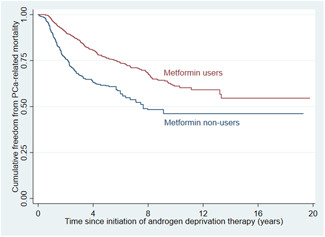
Kaplan–Meier curve showing the cumulative freedom from prostate cancer (PCa)‐related mortality. [Color figure can be viewed at wileyonlinelibrary.com]

**Figure 3:**
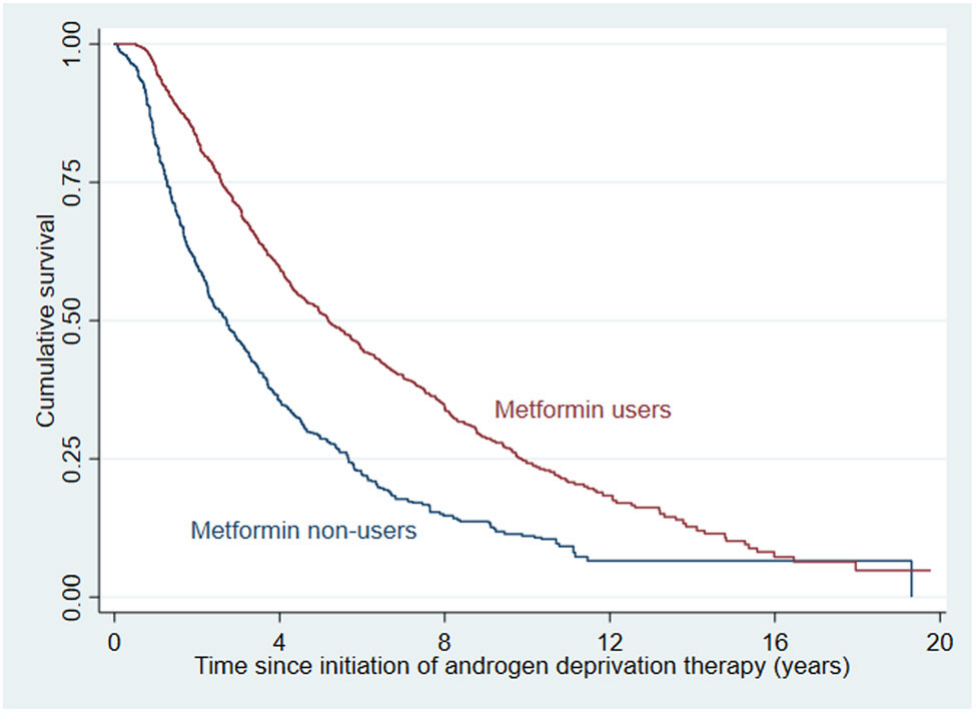
Kaplan–Meier curve showing the cumulative freedom from all‐cause mortality. [Color figure can be viewed at wileyonlinelibrary.com]

Overall, metformin users had significantly lower risks of PCa‐related mortality (wHR 0.49 [0.39, 0.61], *p* < 0.001; Figure 2) and all‐cause mortality (wHR 0.53 [0.46, 0.61], *p* < 0.001; Figure 3).

### 3.3 Subgroup analyses

Among patients with or without AR antagonists or chemotherapy use (*N* = 876 and *N* = 1096, respectively), metformin users had significantly lower risks of PCa‐related mortality and all‐cause mortality, with stronger associations observed in patients without AR antagonist or chemotherapy use (*p* value for interaction = 0.017 and 0.048, respectively; Table 2). This may suggest that the survival benefits associated with metformin may be more pronounced among patients without metastatic PCa.

**Table 2:** Weighted comparisons of outcomes by metformin usage with subgroups for androgen receptor antagonist or chemotherapy usage. Hazard ratios were referenced against metformin non‐users.

	Never received androgen receptor antagonist or chemotherapy (*N* = 1096)		Received androgen receptor antagonist or chemotherapy (*N* = 876)		*p* Value for interaction
	Weighted hazard ratio [95% CI]	*p* Value	Weighted hazard ratio [95% CI]	*p* Value	
Prostate cancer‐related mortality	0.38 [0.27, 0.52]	<0.001	0.59 [0.43, 0.81]	0.001	0.017
All‐cause mortality	0.48 [0.40, 0.57]	<0.001	0.60 [0.48, 0.75]	<0.001	0.048

Metformin users had significantly lower risks of PCa‐related mortality and all‐cause mortality among those who received bilateral orchidectomy only (*N* = 652) or medical castration only (*N* = 1090), as summarized in Supporting Information: Table 5. There were numerical trends for lower risks of PCa‐related mortality and all‐cause mortality for patients who received both bilateral orchidectomy and medical castration; the statistical significance of which may have been strongly dampened by the small number of patients in this subgroup (*N* = 229).

Among both users of insulin (*N* = 501) and non‐users (*N* = 1471), metformin users had significantly lower risks of PCa‐related mortality and all‐cause mortality (*p* value for interaction = 0.642 and 0.384, respectively; Supporting Information: Table 6). Similarly, among both patients with baseline HbA1c level >7% (*N* = 752) and ≤7% (*N* = 1220), metformin users had significantly lower risks of PCa‐related mortality (*p* value for interaction = 0.265) and all‐cause mortality (*p* value for interaction = 0.831), as summarized in Supporting Information: Table 7. These suggested that baseline diabetic control did not affect the observed associations between metformin use and risks of outcomes.

### 3.4 Sensitivity analyses

Sensitivity analysis performed for patients without CKD (*N* = 1828) showed that metformin use remained significantly associated with lower risks of all outcomes (*p* < 0.001 for all; Supporting Information: Table 8). Additionally, metformin use remained significantly associated with lower risks of all outcomes when metformin users were compared only against patients who never used metformin (*N* = 1630; *p* < 0.001 for all; Supporting Information: Table 9), as well as when only metformin users who had metformin use at the time of ADT initiation were compared against patients who never used metformin (*N* = 1535; *p* < 0.001 for all; Supporting Information: Table 10). These suggested that the aforementioned associations were not confounded by CKD, metformin exposure that was not concurrent with ADT, nor metformin use at the time of ADT initiation.

Backward stepwise Cox regression with metformin use duration (years) as a continuous variable showed that longer metformin use duration was associated with lower risks of PCa‐related mortality (HR 0.90 [0.88, 0.92], *p* < 0.001) and all‐cause mortality (HR 0.91 [0.89,0.92], *p* < 0.001), as visualized in Supporting Information: Figure 1 and 2, respectively.

Univariable competing‐risk regression with non‐PCa‐related mortality as the competing event demonstrated robustness of our findings, with metformin use being associated with lower risks of PCa‐related mortality (SHR 0.61 [0.49, 0.76], *p* < 0.001).

## 4 DISCUSSION

This retrospective cohort study showed that, over a mean follow‐up duration of more than 4 years, concurrent metformin use and ADT in Asian, diabetic patients with PCa was associated with significantly lower risks of PCa‐related mortality and all‐cause mortality. Such associations were independent of diabetic control and metformin use nonconcurrent with ADT and appeared to be stronger in those without concurrent use of AR antagonist or chemotherapy. To the best of the authors' knowledge, this was one of the first studies demonstrating survival benefits associated with metformin use concurrent with ADT among patients with PCa in Asia.

### 4.1 Underlying mechanisms

Metformin's anticancer activity may be related to AMP‐activated protein kinase (AMPK) activation. Generally, AMPK can cause cell cycle arrest by inhibiting the protein kinase B/mammalian target of rapamycin signaling pathway^15^ and p70S6 kinase, a downstream target.^16^ Nonetheless, the role of AMPK in PCa is not fully understood. It is hypothesized that each unique AMPK complex regulates downstream processes that can be tumor suppressive or oncogenic, and their weighted net function then determines AMPK's final output, influenced by additional prostate‐specific signaling. ^17^ Furthermore, how metformin inhibits cancer growth is controversial. Metformin may target the lysosomal AMPK pathway through presenilin enhancer 2.^18^ On the other hand, metformin may activate the AMPK/autophagy pathway.^19^ Moreover, AMPK activation can be adaptive for cancer cells under stress.^20^ Metformin's anticancer activity can also be AMPK‐independent. For instance, it may be attributed to its interaction with pathways specific for PCa cell lines. AR signaling is involved in the development of PCa irrespective of castration^21^ by disrupting cell cycle regulation^22^ and activating the erythroblast transformation specific (ETS) oncogene family through ERG.^23^, ^24^ While ADT suppresses AR signaling, ectopic expression of the c‐Myc oncogene could attenuate such effects,^25^ leading to castration‐resistant prostate cancer (CRPC).^26^ In PCa cells treated by metformin, c‐Myc protein levels are reduced and AR signaling could be suppressed.^27^ Additionally, metformin could inhibit androgen‐dependent upregulation of insulin‐like growth factor receptor type I^28^ responsible for promoting the survival, development, and proliferation of PCa cells.^29^

### 4.2 Prior studies and future directions

The association between metformin use and mortality risks in patients with PCa is unclear. While some studies suggested an association between metformin use and improved survival in diabetic patients with PCa,^7^, ^8^ others did not observe any significant associations.^30^, ^31^ Donata et al.^31^ found null association in a nondiabetic population, but their study was limited by a small (*N* = 254) and largely heterogeneous population. Kaushik et al.^30^ also showed null association, but their study was limited to patients with PCa who had undergone radical prostatectomy. Furthermore, PCa‐related mortality was not explored. Their incidence of all‐cause mortality (7.8%) was also much lower than in our study (62.4%), suggesting that our study may be better powered to examine the associations between metformin use and mortality risks. Recently, the protective role of metformin in PCa has been called into question again as contradictive results were shown in prospective trials,^32^, ^33^ whereby the addition of metformin to docetaxel or abiraterone did not suppress PCa progression in patients with metastatic CRPC in the TAXOMET trial (NCT01796028)^32^ and MetAb‐Pro trial (NCT01677897),^33^ respectively. Nonetheless, the TAXOMET trial examined a nondiabetic population, which is inherently different from our study design. In addition, high‐grade diseases were not well‐balanced between the two treatment arms in the TAXOMET trial. Meanwhile, the result of the MetAb‐Pro trial must be interpreted with caution, as metformin was studied in patients who failed abiraterone treatment, which may dilute the survival benefits associated with metformin use. In addition, since the effects of AMPK activation could depend on stimulus duration,^17^ a shorter cumulative metformin exposure in both trials may explain the null results. Metformin users in this study, on the other hand, had a longer exposure to metformin. Since both trials only studied patients with CRPC, the survival benefits brought by metformin's potential role in delaying CRPC may not be shown. Further research is needed to ascertain the survival benefits associated with metformin use and whether they differ between PCa subtypes.

Our results were consistent with metformin's potential role in delaying the development of CRPC. Patients receiving long‐term ADT have higher risks of developing CRPC.^34^ We observed stronger associations between metformin use and mortality risks among patients who never received AR antagonists or chemotherapy, which are typical treatments for metastatic PCa.^35^ The survival benefits associated with metformin use appeared stronger in those without metastatic PCa as they are less likely to had already developed CRPC.^36^ The anticancer activity of metformin was previously demonstrated in patients with localized PCa.^37^ However, clinical evidence in patients with metastatic hormone‐sensitive PCa is lacking. Future clinical studies exploring the potential role of in delaying CRPC in this population is warranted and may supplement current treatment guidelines.

We built on existing literature by showing the associations between metformin use and mortality risks in Asian, diabetic patients with PCa. By studying patients who were receiving ADT, this study extends the generalizability of the survival benefits associated with metformin use to more advanced PCa. These findings were consolidated by subgroup and sensitivity analyses showing that the associations remained significant across different types of ADT and were independent of diabetic control and metformin use nonconcurrent with ADT. Nonetheless, it is unclear whether these associations would remain in nondiabetic patients, as metformin use and lifestyle changes may improve survival by preventing metabolic syndrome caused by androgen suppression.^38^ Although our findings support the potential role of metformin as adjuvant therapy for PCa, further studies are needed.

### 4.3 Strengths and limitations

This study used a representative population‐based database with long follow‐up duration. Our results are thus likely to be widely generalizable and reflect real‐world practice. Sensitivity analyses using different approaches showed consistent results, indicating robustness. However, several limitations should be noted. First, as an observational study, residual confounding cannot be excluded. Second, since all diagnoses were identified using ICD‐9 codes as recorded by CDARS, the data could not be adjudicated individually. Nonetheless, the data input was performed by the treating physicians, and none of the authors had influence over these inputs. In addition, the coding accuracy of the system was well demonstrated in other studies.^11^, ^13^ Third, cancer staging details were unavailable due to the nature of the data. We addressed this limitation by using antiandrogens or chemotherapy as surrogates for metastatic PCa.

## 5 CONCLUSIONS

Metformin use concurrent with ADT in Asian, diabetic patients with PCa was associated with significantly lower risks of PCa‐related and all‐cause mortality. Such associations appeared stronger in patients without AR antagonist or chemotherapy use and were independent of diabetic control and metformin use nonconcurrent with ADT. Further validation in other Asian cohorts is warranted to explore whether the suggested benefits may be further generalizable.

## AUTHOR CONTRIBUTIONS

All authors contributed to the study conception and design. Material preparation, data collection, and analysis were performed by Yan Hiu Athena Lee, Jeffrey Shi Kai Chan, and Kang Liu. Software was supported by Jeffrey Shi Kai Chan. The first draft of the manuscript was written by Yan Hiu Athena Lee, Jeremy Man Ho Hui, and Jeffrey Shi Kai Chan, and all authors commented on previous versions of the manuscript. All authors read and approved the final manuscript. The study was supervised by Gary Tse and Chi Fai Ng.

## CONFLICT OF INTEREST

The authors declare no conflict of interest.

## Supporting information

Supporting information.

Supporting information.

Supporting information.

Supporting information.

Supporting information.

Supporting information.

Supporting information.

Supporting information.

Supporting information.

Supporting information.

Supporting information.

Supporting information.

Supporting information.

## Data Availability

The authors had full access to all the data in the study and take responsibility for the integrity of the data and the accuracy of the data analysis.
