# Supplementary figures and images for: Metformin use and mortality in Asian, diabetic patients with prostate cancer on androgen deprivation therapy: A population‐based study
Source: Prostate. 2022 Sep 30;83(1):119–27. doi: 10.1002/pros.24443 (PMC9742285; doi:10.1002/pros.24443)

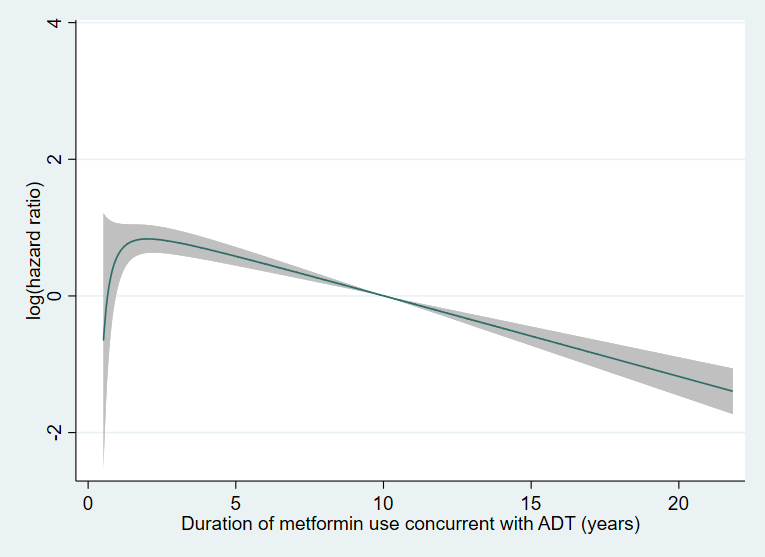

Supplement: Supplementary file 1 — Supporting information. [file PROS-83-119-s001.tif]

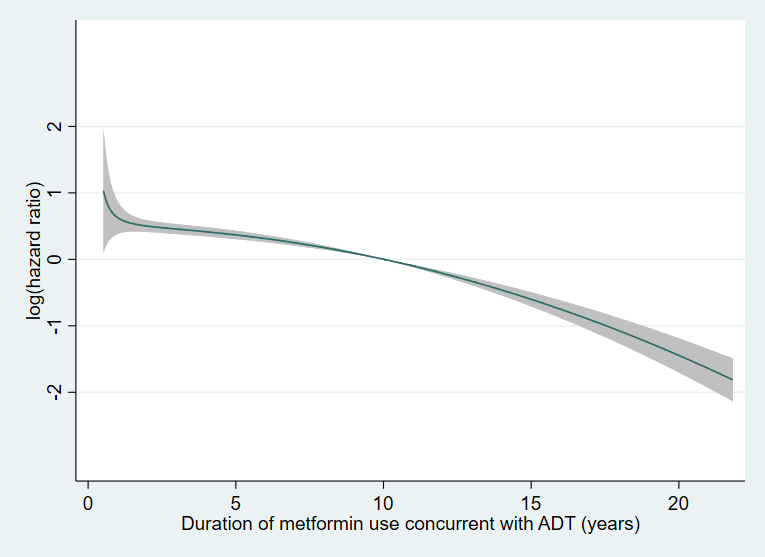

Supplement: Supplementary file 2 — Supporting information. [file PROS-83-119-s009.tif]
