# Supplementary material for: Metformin use and mortality in Asian, diabetic patients with prostate cancer on androgen deprivation therapy: A population‐based study
Source: Prostate. 2022 Sep 30;83(1):119–27. doi: 10.1002/pros.24443 (PMC9742285; doi:10.1002/pros.24443)
Supplement: Supplementary file 3 — Supporting information. [file PROS-83-119-s008.docx]

**Supplementary figure 1.** Fractional polynomial curve showing the association between metformin use duration and prostate cancer-related mortality.

**Supplementary figure 2.** Fractional polynomial curve showing the association between metformin use duration and all-cause mortality.
