# Supplementary material for: Metformin use and mortality in Asian, diabetic patients with prostate cancer on androgen deprivation therapy: A population‐based study
Source: Prostate. 2022 Sep 30;83(1):119–27. doi: 10.1002/pros.24443 (PMC9742285; doi:10.1002/pros.24443)
Supplement: Supplementary file 4 — Supporting information. [file PROS-83-119-s003.docx]

**Supplementary Table 1.** *International Classification of Diseases, Ninth Revision* (ICD-9) and *International Classification of Diseases, Tenth Revision* (ICD-10) codes used to identify outcomes and co-morbidities. All hereby listed codes include the corresponding sub-codes.

| Prostate cancer-related mortality | ICD-9: 185  ICD-10: C61 |
| --- | --- |
