# Supplementary material for: Metformin use and mortality in Asian, diabetic patients with prostate cancer on androgen deprivation therapy: A population‐based study
Source: Prostate. 2022 Sep 30;83(1):119–27. doi: 10.1002/pros.24443 (PMC9742285; doi:10.1002/pros.24443)
Supplement: Supplementary file 6 — Supporting information. [file PROS-83-119-s006.docx]

**Supplementary Table 3.** List of medications.

| ACEI/ARB |
| --- |
| Beta-blockers |
| Dihydropyridine calcium channel blockers |
| Insulin |
| Statins |
| Corticosteroids |
| Antiplatelets |
| Anticoagulants |
| Androgen receptor antagonists (Enzalutamide, Bicalutamide, Abiraterone) |
| Chemotherapeutic agents (Docetaxel, Cabazitaxel, Estramustine, Mitoxantrone) |

ACEI, angiotensin converting enzyme inhibitor. ARB, angiotensin receptor blocker.
