# Supplementary material for: Metformin use and mortality in Asian, diabetic patients with prostate cancer on androgen deprivation therapy: A population‐based study
Source: Prostate. 2022 Sep 30;83(1):119–27. doi: 10.1002/pros.24443 (PMC9742285; doi:10.1002/pros.24443)
Supplement: Supplementary file 7 — Supporting information. [file PROS-83-119-s007.docx]

**Supplementary Table 4**. Differences in baseline characteristics between patients included and excluded in the analysis

|  | Included (N=1971) | Excluded (N=915) |
| --- | --- | --- |
| Age, years | 76.2±7.8 | 76.3±8.3 |
| Use of GnRH agonist or antagonist, n (%) | 1319 (66.9) | 616 (67.3) |
| Bilateral orchidectomy, n (%) | 881 (44.7) | 358 (39.1) |
| Hypertension, n (%) | 995 (50.5) | 410 (44.8) |
| Ischaemic heart disease, n (%) | 416 (21.1) | 140 (15.3) |
| Myocardial infarction, n (%) | 122 (6.2) | 43 (4.7) |
| Heart failure, n (%) | 192 (9.7) | 78 (8.5) |
| Stroke or transient ischaemic attack, n (%) | 296 (15.0) | 119 (13.0) |
| Chronic kidney disease, n (%) | 144 (7.3) | 55 (6.0) |
| Anaemia, n (%) | 219 (11.1) | 93 (10.2) |
| Atrial fibrillation, n (%) | 133 (6.8) | 67 (7.3) |
| Chronic liver disease, n (%) | 44 (2.2) | 19 (2.1) |
| Chronic obstructive pulmonary disease, n (%) | 97 (4.9) | 60 (6.6) |
| Hyperlipidaemia, n (%) | 437 (22.2) | 162 (17.7) |
| Ever underwent radiotherapy, n (%) | 353 (17.9) | 204 (22.3) |
| Ever underwent radical prostatectomy, n (%) | 638 (32.4) | 309 (33.8) |
| Any malignancy, n (%) | 255 (12.9) | 123 (13.4) |
| ACEI/ARB use, n (%) | 1223 (62.1) | 3982 (42.8) |
| Beta-blocker use, n (%) | 974 (49.4) | 360 (39.3) |
| Dihydropyridine calcium channel blocker use, n (%) | 1288 (65.4) | 490 (53.6) |
| Insulin use, n (%) | 501 (25.4) | 221 (24.2) |
| Statin use, n (%) | 1181 (59.9) | 344 (37.6) |
| Corticosteroid use, n (%) | 356 (18.1) | 188 (20.6) |
| Antiplatelet use, n (%) | 776 (39.4) | 316 (34.5) |
| Anticoagulant use, n (%) | 115 (5.8) | 48 (5.3) |
| Androgen receptor antagonist use, n (%) | 835 (42.4) | 259 (28.3) |
| Prior chemotherapy, n (%) | 11 (0.6) | 1 (0.1) |
| Chemotherapy concurrent with ADT, n (%) | 165 (8.4) | 43 (4.7) |
| HbA1c, % | 7.0±1.3 | 7.0±1.3 ^1^ |

ACEI, angiotensin converting enzyme inhibitor. ADT, androgen deprivation therapy. ARB, angiotensin receptor blocker. GnRH, gonadotropin hormone-releasing hormone. HbA1c, haemoglobin A1c.

^1^ Available for 444 patients (48.5%)
