# Supplementary material for: Metformin use and mortality in Asian, diabetic patients with prostate cancer on androgen deprivation therapy: A population‐based study
Source: Prostate. 2022 Sep 30;83(1):119–27. doi: 10.1002/pros.24443 (PMC9742285; doi:10.1002/pros.24443)
Supplement: Supplementary file 8 — Supporting information. [file PROS-83-119-s010.docx]

**Supplementary Table 5.** Weighted comparisons of outcomes by metformin usage with subgroups for the type of androgen deprivation therapy. Hazard ratios were referenced against metformin non-users.

|  | Bilateral orchidectomy only (N=652) | | GnRH agonist or antagonist only (N=1090) | | GnRH agonist or antagonist followed by bilateral orchidectomy (N=229) | |
| --- | --- | --- | --- | --- | --- | --- |
|  | Weighted hazard ratio [95% confidence interval] | p value | Weighted hazard ratio [95% confidence interval] | p value | Weighted hazard ratio [95% confidence interval] | p value |
| Prostate cancer-related mortality | 0.37 [0.27, 0.52] | <0.001 | 0.59 [0.43, 0.82] | 0.001 | 0.67 [0.38, 1.18] | 0.166 |
| All-cause mortality | 0.48 [0.38, 0.61] | <0.001 | 0.50 [0.42, 0.61] | <0.001 | 0.74 [0.50, 1.11] | 0.148 |

GnRH, gonadotropin hormone-releasing hormone.
