# Supplementary material for: Metformin use and mortality in Asian, diabetic patients with prostate cancer on androgen deprivation therapy: A population‐based study
Source: Prostate. 2022 Sep 30;83(1):119–27. doi: 10.1002/pros.24443 (PMC9742285; doi:10.1002/pros.24443)
Supplement: Supplementary file 9 — Supporting information. [file PROS-83-119-s004.docx]

**Supplementary Table 6.** Weighted comparisons of outcomes by metformin usage with subgroups for insulin use. Hazard ratios were referenced against metformin non-users.

|  | Users of insulin (N=501) | | Non-users of insulin (N=1471) | | p value for interaction |
| --- | --- | --- | --- | --- | --- |
|  | Weighted hazard ratio [95% confidence interval] | p value | Weighted hazard ratio [95% confidence interval] | p value |  |
| Prostate cancer-related mortality | 0.54 [0.36, 0.81] | 0.003 | 0.48 [0.37, 0.62] | <0.001 | 0.642 |
| All-cause mortality | 0.59 [0.46, 0.76] | <0.001 | 0.51 [0.43, 0.61] | <0.001 | 0.384 |
