# Supplementary material for: Metformin use and mortality in Asian, diabetic patients with prostate cancer on androgen deprivation therapy: A population‐based study
Source: Prostate. 2022 Sep 30;83(1):119–27. doi: 10.1002/pros.24443 (PMC9742285; doi:10.1002/pros.24443)
Supplement: Supplementary file 10 — Supporting information. [file PROS-83-119-s012.docx]

**Supplementary Table 7.** Weighted comparisons of outcomes by metformin usage with subgroups for HbA1c level. Hazard ratios were referenced against metformin non-users.

|  | HbA1c >7% (N=752) | | HbA1c ≤7% (N=1220) | | p value for interaction |
| --- | --- | --- | --- | --- | --- |
|  | Weighted hazard ratio [95% confidence interval] | p value | Weighted hazard ratio [95% confidence interval] | p value |  |
| Prostate cancer-related mortality | 0.43 [0.30, 0.62] | <0.001 | 0.55 [0.42, 0.71] | <0.001 | 0.265 |
| All-cause mortality | 0.52 [0.41, 0.67] | <0.001 | 0.53 [0.45, 0.62] | <0.001 | 0.831 |

HbA1c, hemoglobin A1c.
