# Supplementary material for: Metformin use and mortality in Asian, diabetic patients with prostate cancer on androgen deprivation therapy: A population‐based study
Source: Prostate. 2022 Sep 30;83(1):119–27. doi: 10.1002/pros.24443 (PMC9742285; doi:10.1002/pros.24443)
Supplement: Supplementary file 11 — Supporting information. [file PROS-83-119-s013.docx]

**Supplementary Table 8.** Sensitivity analysis of patients without chronic kidney disease (N=1828). Weighted comparisons of outcomes by metformin usage were presented. Hazard ratios were referenced against metformin non-users.

|  | Weighted hazard ratio [95% confidence interval] | p value |
| --- | --- | --- |
| Prostate cancer-related mortality | 0.49 [0.39, 0.62] | <0.001 |
| All-cause mortality | 0.51 [0.44, 0.59] | <0.001 |
