# Supplementary material for: Metformin use and mortality in Asian, diabetic patients with prostate cancer on androgen deprivation therapy: A population‐based study
Source: Prostate. 2022 Sep 30;83(1):119–27. doi: 10.1002/pros.24443 (PMC9742285; doi:10.1002/pros.24443)
Supplement: Supplementary file 13 — Supporting information. [file PROS-83-119-s011.docx]

**Supplementary Table 10.** Sensitivity analysis excluding patients who had any metformin exposure from the non-user group (total N=1535) and including only patients who had metformin exposure at the time of androgen deprivation therapy initiation as the user group. Weighted comparisons of outcomes by metformin usage were presented. Hazard ratios were referenced against metformin non-users.

|  | Weighted hazard ratio [95% confidence interval] | p value |
| --- | --- | --- |
| Prostate cancer-related mortality | 0.43 [0.32, 0.57] | <0.001 |
| All-cause mortality | 0.50 [0.41, 0.60] | <0.001 |
